# TP-RotatE: A knowledge graph representation learning method combining path information and rules to capture complex relational patterns

**DOI:** 10.1371/journal.pone.0324059

**Published:** 2025-05-27

**Authors:** Xinliang Liu, Yanyan Shi, Yushi Xu, Yanzhao Ren

**Affiliations:** 1 School of Computer and Artificial Intelligence, Beijing Technology and Business University, Beijing, China; 2 Nation Engineering Research Center for Agri-product Quality Traceability, Beijing Technology and Business University, Beijing, China; Air Force Engineering University, CHINA

## Abstract

Representation learning on a knowledge graph (KG) aims to map entities and relationships into a low-dimensional vector space. Traditional methods for representation learning have predominantly focused on the structural aspects of triples within the KG. While existing approaches have endeavored to integrate path information and rules to enhance the structural richness of KGs, these efforts have been constrained by the lack of consideration for complex relational representations and contextual information. In this study, we introduce TP-RotatE, an innovative method that leverages the semantic context of triples to effectively capture more intricate relational patterns. Specifically, our model harnesses contextual information surrounding the head entity and distills relevant rules. These rules are then integrated with path information to offer a more holistic perspective on the relationships embedded within complex vector spaces. Furthermore, the synergy between rules and paths empowers the knowledge-embedded model to handle the intricacies of complex relationships. Experimental results on a benchmark dataset confirm that TP-RotatE surpasses current baseline methods in KG inference tasks, achieving state-of-the-art performance.

## 1. Introduction

A knowledge graph (KG) is a system that gathers and amalgamates information into an ontology and employs a reasoner to deduce new insights [1]. It consolidates various extensive knowledge bases, such as Freebase [2] and AI-KG [3]. Typically, a KG is composed of numerous triples (*h, r, t*) that encode factual data, where *h*, *r*, and *t* denote the head entity, relation, and tail entity, respectively. Given its capacity to efficiently deliver structured information, KGs are extensively utilized in a range of downstream applications, including intelligent question answering [4], personalized recommendations [5], and information retrieval [6]. However, while large-scale KGs may encompass billions of triples, the knowledge they contain represents only a small fraction of the total real-world information and may also harbor inaccuracies and inconsistencies. For instance, 71% of individuals in Freebase lack documented birthplaces, and 75% have no assigned nationality [7]. Recent research has also explored extensively the adversarial robustness of knowledge representations. Tian [8] highlights the potential vulnerabilities in structured knowledge representations that leverage adversarial machine learning techniques to generate covert false data attacks. These studies further illustrate the challenges and progress made in KGR. Such gaps in information can adversely impact downstream tasks. To counter these limitations, the field of knowledge graph reasoning (KGR) has emerged. KGR endeavors to employ reasoning over KGs to surmount existing knowledge gaps and deduce missing elements within the KG.

Recent approaches to knowledge graph completion predominantly rely on embedding techniques, which map entities and relationships into low-dimensional vector spaces [9]. Notable examples in this domain include TransE [10] and TransH [11]. However, single-triple inference methods often struggles with sparse data, while multi-step paths can provide more comprehensive relational information. For instance, research by Seo et al. [12] and Lin et al. [13] seeks to develop more nuanced knowledge representations by integrating intermediate entities and relationship representations along relational paths. Since path representations are intrinsically connected to the relationships, and considering the variety of relationship types in knowledge bases—such as symmetric relationships (e.g., alliances), antisymmetric relationships (e.g., parent-child), inverse relationships (e.g., employer-employee), and compositional relationships (e.g., the head of a company’s R&D department)—current methods for embedding relationships find it challenging to fully encapsulate these intricacies. Furthermore, the dependence on numerical computations for path representations also makes them prone to inaccuracies.

To bolster inference precision and enhance interpretability, the incorporation of logical rules is recognized as a potent strategy for enriching sparse knowledge graphs (KGs). Niu et al. [14] and Niu et al. [15] have demonstrated the successful integration of Horn clauses into triples and path embeddings, thereby amplifying the efficacy of representation learning. However, the efficacy of automated rule extraction in sparse KGs is often limited, especially for targeted queries, as the challenge of pinpointing contextually relevant rules remains a formidable obstacle.

In this research, we introduce TP-RotatE, a groundbreaking combinatorial representation learning model that integrates head entity subgraph rules and path information. This model innovatively merges the RotatE embedding approach with path representations, addressing the previous limitations in handling different relationship types and thereby enhancing the precision of relationship embeddings. By harnessing the advanced information interaction capabilities of the Transformer architecture, the model consolidates contextual information around head entities to formulate rules. Once these rules are established in specific formats, they guide the integration of relationships within paths. The TP-RotatE model was subjected to rigorous evaluation across three benchmark datasets. The experimental outcomes not only highlight its exceptional performance in KG completion tasks but also significantly outperform existing baselines, substantiating the efficacy of integrating rules and paths in KG embeddings. The pivotal contributions of this study are as follows:

(1) The incorporation of subgraph rule information centered on head entities, which facilitates the aggregation of subgraph information and the exploitation of contextual cues to bolster e the reasoning process and improves the model’s ability to capture relational structures.
(2) The synergy between RotatE embedded models and paths achieves a more comprehensive representation of relationship types, which enables a more expressive representation of different types of relationships and allows for the merging of more complex relationships. This integration not only captures the various relationship patterns more effectively, but also facilitates their combination to model more complex relationship structures.
(3) The proposal of a knowledge representation learning model that seamlessly integrates relationship path information, logical rule information, and entity and relation information from the RotatE embedding model. Our experiments confirm that this model outperforms all baseline methods, setting a new standard in the field.

## 2. Related work

**Embedding model based on translation mechanism:** In recent years, significant advancements have been made in distributed representations of entities and relations in KG learning, which can be categorized into four primary groups: 1) **Traditional translation embedding models**. Inspired by the word word vector embedding model Word2Vec, TransE interprets relations as translation operations in a low-dimensional embedding space. Specifically, for a valid triple (*h, r, t*), the vector of the head entity combined with the relation vector should approximate the vector of the tail entity, satisfying h+r≈t . However, TransE performs poorly in handling complex relations such as one-to-many or many-to-many. The TransH model addresses this limitation by introducing projection, allowing entities to be represented differently under specific relation types. 2) **Translation embedding models based on neural network**. DistMult [16] simplifies matrix decomposition by restricting bilinear matrices to diagonal matrices. By introducing tensor decomposition in ComplEx [17] for knowledge completion, ComplEx effectively handles numerous binary relations, overcoming the limitation of DistMult in representing symmetric relations. Additionally, Wang [18] utilizes Capsule Networks to model knowledge graphs, capturing hierarchical entity dependencies and improving robustness to noisy data. By leveraging dynamic routing mechanisms, Caps-OWKG effectively represents relational structures and enhances knowledge graph completion performance. 3) **Translation embedding models based on relational paths.** In KGs, entities are connected through indirect relations, which are addressed by multi-step path inference models. While TransE only considers direct relationships, it fails to capture relational paths. PTransE [19] model treats relational paths as transformations between entities, employing the path constraint resource allocation algorithm to evaluate path reliability and representing paths through the recursive combination of relational embeddings. Additionally, Jin [20] explores how rule-based relational patterns can enhance knowledge graph embeddings. By leveraging learned logical rules, this approach captures multi-hop relational dependencies, improving the expressiveness of translation-based models in complex reasoning tasks. Recently, Dong [21] introduces a memory mechanism to dynamically update path representations over time, enhancing temporal knowledge graph reasoning. This approach improves the model’s ability to capture time-dependent multi-hop relations, making it more effective for dynamic knowledge graphs. 4)**Translation embedding models based on geometric relations**. TransF [22] models the relationship between the head and tail entities as a flexible vector translation, avoiding strict transformations based on vector addition. For triples (*h*,*r*,*t*), it relaxes the requirement of h+r≈t, ins*t*ead enforcing h+r≈αt, where α reflects flexibility. This approach maintains consistent embedding directions without requiring vector magnitudes, improving model performance in reflexive, one-to-many, and many-to-one relations.

Despite these significant strides, these methods face limitations due to their exclusive reliance on triplet information. This dependency can result in limited accuracy and diminished interpretability.

**Rule extraction and models enhanced by rules:** The interpretability and semantic richness of logical rules render them indispensable in knowledge reasoning. They empower systems to engage in more profound reasoning and comprehension, thereby enhancing the quality and utility of knowledge graphs (KGs). In the realm of rule learning on KGs, the rule mining method employed by AMIE [23] aims to generate edge relationship rules. It initiates by pre-generating all potential rules predicated on edge types. Subsequently, it discerns instances within the graph that conform to these rules and computes their confidence levels. A rule is deemed valid if its confidence surpasses a predefined threshold. RuleN [24], on the other hand, proposes an efficient framework for rule mining. It systematically identifies paths of length n between the head entity *a* and the tail entity *b* using depth-first search. These paths serve as the rule bodies, which are then utilized to construct the rules.

The essence of traditional symbol-based rule learning methods lies in approximating specific paths derived from graph traversal as rules. This is achieved by searching and exploring paths across the entire graph or a sampled subset. The rule generation process in these algorithms is significantly influenced by both the search algorithms employed and the pruning techniques applied. The choice and optimization of these methods are pivotal for ensuring the quality and efficiency of the rule mining results.

Recently, differentiable rule-learning methods have gained popularity for their ability to simultaneously learn the confidence and structure of rules. Neural LP [25], based on Tensorlog [26], integrates first-order parameter learning with the structural learning of logic rules in an end-to-end microscopic model. Neural-num-LP [27] extends Neural LP by incorporating numerical attributes into rule bodies, enabling operations such as comparisons, aggregations, and negations between entities. Meanwhile, in the field of deep learning, Saeedan et al. [28] proposed Detail-Preserving Pooling (DPP), which enhances traditional pooling methods by preserving critical details while reducing feature dimensions.

In KGs with sparse data, recent studies have explored integrating rules with KG embeddings to enhance data augmentation. Cohen et al. [29] utilized relational embeddings to derive rules and generate new factual triples associated with sparse entities based on these inferred rules. However, this method generates only a limited set of rules due to the restricted relationships in the KG and fails to exploit its inherent semantic information. Li et al. [30] proposed a logically guided semantic representation learning model for zero-sample relation classification. The method establishes implicit and explicit semantic connections between seen and unseen relations through knowledge graph embeddings and logic rules, effectively bridges the gap between seen and unseen relations, and shows significant improvement potential in zero-sample relation classification tasks. Wang et al. [31] enriched the model by incorporating ontological information of entities, enhancing the ability to distinguish between multiple entity classes and improving both accuracy and interpretability. Previous rule mining approaches, which relied on automated rule mining, were inadequate for addressing context-aware information in KGs, an aspect crucial for handling sparse KGs.

## 3. Methodology

**Fig 1:**
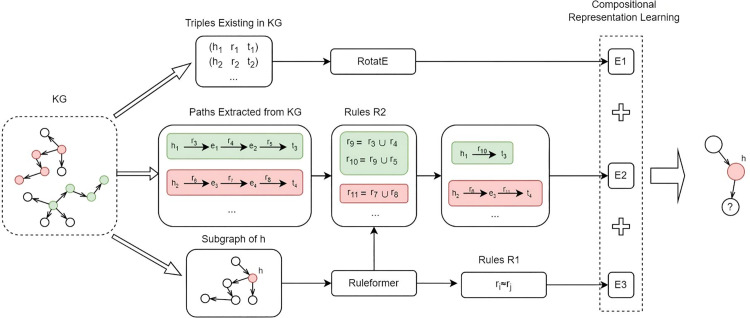
Overall architecture of the model.

This study seamlessly integrates subgraph-based rule mining with relational paths to distill more profound semantic insights from the model. The comprehensive framework of the model is depicted in Fig 1. The process first mines rules of varying lengths based on the contextual information surrounding the head entity. R_1_ and R_2_ represent rules of length 1 and 2, respectively. After that, combined with the paths mined from the knowledge graph, rule R_2_ is used to synthesize the paths while rule R_1_ establishes semantic links between specific relational formulas. Concurrently, entities and relations are projected into a vector space through vector initialization, facilitating the training of embeddings for the KG. The learning process is designed to refine the representation of triples, paths, and correlation pairs, thereby enhancing their semantic cohesion.

### 3.1. Rule information extraction

**Fig 2:**
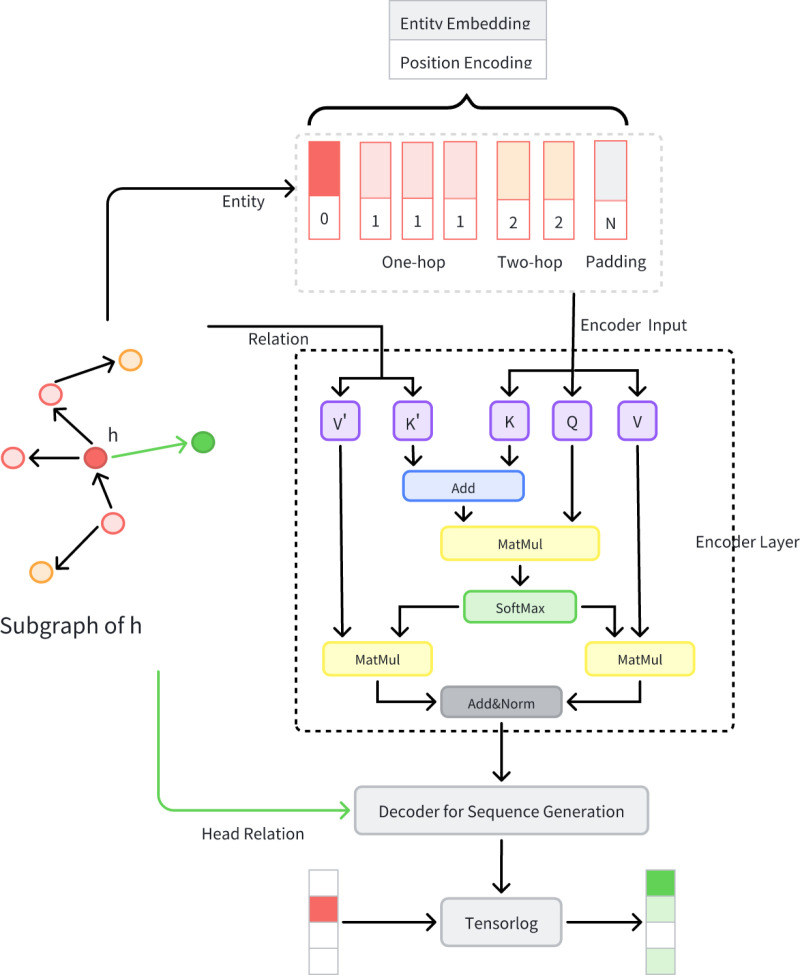
Model framework for rule extraction.

The model is composed of two core components: 1) An encoder block that assimilates subgraph information related to the head entity and facilitates the interaction of contextual information. This component is crucial for capturing the rich contextual nuances that surround the head entity within the knowledge graph; 2) A decoder block that leverages the aggregated entity embeddings to calculate relational probabilities at each stage of rule micromining, as illustrated in Fig 2. This component plays a pivotal role in translating the aggregated information into actionable insights, particularly in the context of rule-based reasoning within the knowledge graph.

For triples (*h,r,t*), the correlation subgraph of the head entity *h* is first extracted, as it potentially contains contextual information about the head entity *h*. *S*_*k*_(*h*) is defined as the subgraph consisting of the *k*-hop neighbors of *h* along with the set of edges connecting these entities. Since *S*_*k*_(*h*) is a graph structure and the Transformer model operates as a seq2seq framework, it is necessary to transform the subgraph into a sequence, denoted as *S*_*node*_=[*e*_*1*_,*e*_*2*_,...*e*_*num*_,...*blank*], where *num* represents the total number of entities surrounding the head entity, and a token *blank* is needed for padding. The positional embedding is then derived by appending the shortest path distance to the central entity *h*.

To enhance representation learning, the type of relationship associated with the entity is incorporated. The representation *x*_*e*_ of an entity *e* is computed by summing the type embedding of the associated relationship with the randomly initialized embedding *y*_*e*_*.* This process is expressed as follows:


$$x_{e}=\underset{i=1}{\overset{\left|R\right|}{\sum }}\;b_{i}^{dom}r_{i}^{dom}+\underset{i=1}{\overset{\left|R\right|}{\sum }}\;b_{i}^{ran}r_{i}^{ran}+y_{e}$$ (1)

Here, *rdom i*and *rran i*denote the domain and range embeddings of the relation *r*_*i*_, respectively. The parameters *bdom i*and *bran i* are normalized to account for the diversity of distinct relation types. This normalization is expressed as follows:


$$b_{i}^{dom}=\frac{n_{i}^{dom}}{\sum_{j=1}^{\left|R\right|}n_{j}^{dom}},b_{i}^{ran}=\frac{n_{i}^{ran}}{\sum_{j=1}^{\left|R\right|}n_{j}^{ran}}$$ (2)

where *ndom i*and *n*ran i represent the number of relationships of each type connected to or associated with *e*. Accordingly, the sequence of nodes in the subgraph can be expressed as *S*_*e*_= [*x*_1_*, x*_2_*,...x*_*num*_*,...blank*]. Building on this, attention mechanisms are introduced to incorporate edge information. Similar to embedding relational information into entity representations, relationship data are integrated into the value computation step:


$$z_{i}=\underset{j=1}{\overset{n}{\sum }}\;a_{ij}\left(x_{j}W^{V}+\underset{r=1}{\overset{\left|R\right|}{\sum }}\;k_{r}x_{r}W^{V^{\prime }}\right)$$ (3)

where *W*^V^ represents the entity value matrix, and *W^V′^* denotes the relational value matrix.

After the encoder outputs the entity sequence *S*_e_*^′^*, the decoder combines the encoded information with the head entity and iteratively generates the sequence until the rule sequence *S*_r_ with a rule length of *T* is produced. This process constitutes the rule mining performed by the model.

Specifically, the head relation *x*_r_ is embedded as the starting element of the rule sequence *S*_r_ in the decoder input, i.e., S0 r = x_r_. Through cross-computation of *S*_r_ and *S*_e_*^′^*, the decoder obtains the vector for the subsequent relation. After MLP deployment, the probability ω*i t* corresponding to relation *r*_i_ at step *t* is derived. During inference, the relation with the highest probability is selected to complete the remaining sequence.


$$w_{t+1}=MLP\left(CrossAttention\left(S_{r}^{t},S_{e}^{\prime }\right)\right)$$ (4)

In the inference process, *ω*_*t+1*_ ∈ *R*^*|R|×1*^ denotes the probability distribution over all possible relations at step *t.* Let *r*_*t+1*_ represent the relation with the highest probability among them. Based on this, $S_{r}^{t+1}=\left[S_{r}^{t},x_{r_{t+1}}\right]$ can be determined. This procedure is iterated *T* times to construct a complete rule set of length *T*.

Using this method, rules of lengths 1 and 2 are derived and encapsulated into the respective rule sets, denoted as *R*_*1*_ and *R*_*2*_.

For the rule set of length 1, it is assumed that when the rule holds true, the semantic value of the relation *r*_*1*_ in the rule set is greater than that of the direct relation *r*_*2*_ between entities. Furthermore, the embedding representation of a pair of relations in a rule set exhibits higher semantic similarity than that of two non-contiguous relations. It should be noted that in representation learning, the rule $\left(a,r_{2},b\right)=\left(b,r_{1},a\right)$ should be represented as $\left(a,r_{2},b\right)=\left(a,r_{1}^{-1},b\right)$.

For the rule set of length 2, the previous rule generation model is applied to derive rules for the following pattern:


$$\mathrm{\backslash [}r\left(A,B\right)\leftarrow r_{1}\left(A,C_{1}\right)\wedge \ldots \wedge r_{T}\left(C_{T-1},B\right)\mathrm{\backslash ]}$$

Let *T* denote the length of the rule. Here, *r* and *r_i_* represent relations within the set R. The variables *A*, *B*, and *C*_*i*_ act as placeholders that can be substituted with specific entities. The expression *r* (*A, B*) denotes a rule in the form of a triple. The segment of the triple to the left of the arrow is referred to as the rule head, while the segment to the right is identified as the rule body. For the rule set *R*_*2*_, the rules are encoded to represent a directed path corresponding to the rule body. This path is sequentially constructed from the atoms of each rule body. By encoding these eight rules, a valid path set *P* (*h, t*) is established for the entity pair (*h, t*). The encoding rules are shown in Table 1.

**Table 1:** R_2_ lists the transformation modes.

Original Rules^a^	Encoder Rules^b^
r_3_ (a, b)=r_1_ (a, e) ∪ r_2_ (e, b)	r_3_ = r_1_∪r_2_
r_3_ (a, b)=r_1_ (e, b) ∪ r_2_ (a, e)	r_3_ = r_2_∪r_1_
r_3_ (a, b)=r_1_ (e, b) ∪ r_2_ (e, a)	r_3_ = r_-1 2_∪r_1_
r_3_ (a, b)=r_1_ (e, a) ∪ r_2_ (e, b)	r_3_ = r_-1 1_∪r_2_
r_3_ (a, b)=r_1_ (a, e) ∪ r_2_ (b, e)	r_3_ = r_2_∪r_-1 2_
r_3_ (a, b)=r_1_ (b, e) ∪ r_2_ (a, e)	r_3_ = r_2_∪r_-1 1_
r_3_ (a, b)=r_1_ (e, a) ∪ r_2_ (b, e)	r_3_ = r_-1 1_∪r_-1 2_
r_3_ (a, b)=r_1_ (b, e) ∪ r_2_ (e, a)	r_3_ = r_-1 2_∪r_-1 1_

^a^The left side shows the original rule extracted from the KG.

^b^The right side shows the encoded rule.

To harness the encoded rules effectively, paths must be traversed semantically, iteratively combining operations until no further relationships can be merged. This process encompasses two primary scenarios: (1) In the ideal case, all relationships along the path can be sequentially combined using rule R_2_, culminating in a single relationship that connects the entity pairs. (2) More commonly, some relationships cannot be directly formed based on rule R_2_, necessitating numerical operations, such as addition, to embed these relationships into the model. Furthermore, when multiple rules can be matched along a path simultaneously—for instance, when both and are activated—the rule with the highest confidence is selected to guide the combination of relationships. This approach ensures that the most reliable rule influences the embedding process, there by enhancing the accuracy of the knowledge graph.

### 3.2. Relational path modeling

In this section, the model component utilizing paths is introduced, focusing on the representations of entities and relationships encompassing three types of relations. For a KG comprising a set of triples *S*= {(*h, r, t*)}, the composite embedding capabilities of the head entity *h* and the tail entity *t* are mapped, where *h, t* ∈*C*_*k*_*, r* denotes the relationship linking the two entities. When the relationship is valid, the model is designed to yield a low energy score; otherwise, a high energy score is expected.

For every triplet (*h, r, t*), Rotate represents the element-wise rotation induced by the relation *r*, mapping the head entity *h* to the tail entity *t*. The scoring function is defined as:


$$\begin{array}{c} E\left(h,r,t\right)=∥h\circ r-t∥ \end{array}$$ (5)

Here, ○ represents Hadamard product. For sparse data, the training samples in the KG are often limited, making it difficult for models to accurately identify precise connections between them. Incorporating path information enables the linkage of long-tail entities and relationships with others, thereby facilitating the extraction of richer and more accurate inferences from the data.

The path between entities *h* and *t* is represented as *p* (*r*_*1*_*, r*_*2*_*.... r*_*n*_), and there may be multiple such paths. The triplet score *E* (*h, P, t*), which accounts for multi-step relationships, is defined as:


$$E\left(h,P,t\right)=\frac{1}{Z}\underset{p\in P\left(h,t\right)}{\sum }\;R\left(p|h,t\right)E\left(h,p,t\right),$$ (6)

*R* (*p| h, t*) represents the reliability of the connection pathway between entities *h* and *t*. $Z=\sum {\mathrm{\backslash nolimits}}_{p\in P\left(p|h,t\right)}R\left(p|h,t\right)$, acting as a normalization coefficient, is combined with *E* (*h, p, t*), which denotes the energy function of the triplet (*h, p, t*).

The Path-Constrained Resource Allocation (PCRA) algorithm is employed to evaluate the reliability of relational pathways. Specifically, for the path triplet (*h, p, t*), the pathway *p*= (*r*_*1*_*, r*_*2*_*...r*_*l*_) from the starting entity *h* to the terminal entity *t* is determined, and the pathway can be expressed as $E_{0}\overset{r1}{\to }\;E_{1}\overset{r2}{\to }\;\ldots \overset{rl}{\to }\;E_{l}$, where *h* ∈ *E*_*0*_, and *t* ∈ *E*_*l*_. For any entity *m* ∈ *E*_*i*_, its preceding edge along rela*t*ion *r*_*i*_ is denoted as *B*_*i*_ (*m*), and its succeeding edge along relation *r*_*i*_ is represented as *C*_*i*_ (*m*). The resources directed to m are defined as follows:


$$R\left(m\right)=\underset{n\in B_{i}\left(m\right)}{\sum }\;\frac{1}{|C_{i}\left(n\right)|}R\left(n\right)$$ (7)

where *R*(*n*) represents the resources derived from entity *n*. For each relational path, the initial resource is set as *R* (*h*) =1, and resources are iteratively allocated along the path to determine the resource allocation *R* (*t*) of the tail entity. The reliability of the relational path is represented by the resource obtained by the tail entity, *R* (*t*) =*R* (*p*|*h,t*).

To compute the energy function of the path triplet (*h, p, t*), a method analogous to the scoring function in the RotatE triplet is employed. For the path *p*= (*r*_*1*_*,... r*_*l*_), the composite operation is defined as follows to obtain the path embedding.


$$p=r_{1}+\ldots +r_{l}$$ (8)

### 3.3. Compositional representation modeling

For every triplet (*h, r, t*), this study defines the following energy functions, which establish modular dependencies for triplets of path pairs based on rules *R*_*2*_ and *R*_*1*_.


$$E_{1}\left(h,r,t\right)=\left|\left|hr-t\right|\right|,$$ (9)

$$E_{2}\left(p,r\right)=R\left(p\;\;|\;\;h,t\mathrm{\backslash rightleft}(\underset{{\;\;\mu \;\;}_{i}\in U\left(p\right)}{\prod }\;{\;\;\mu \;\;}_{i}\right)\left|\left|H\left(p\right)-r\right|\right|,$$ (10)

$$E_{3}\left(r,r_{e}\right)=\left|\left|r-r_{e}\right|\right|,$$ (11)

The energy function *E*_*1*_ represents the triplet score. *E*_*2*_ denotes the energy function used to evaluate the similarity between path *p* and relation *r*. The confidence level set *U*(*p*)=*μ*_*1*_*,... μ*_*n*_ corresponds to all rules in the rule set *R*_*2*_ applied during the formation of path *p*. *E*_*3*_(*r, r*_*e*_) characterizes the relationship between relation *r* and *r*_*e*_. If *r*_*e*_ can be associated with relation *r* in the triplet through rule *R*_*1*_, *E*_*3*_ is expected to assign it a lower value.

### 3.4. Loss function

The loss function is defined as a fractional function based on margins, specifically designed for the purpose of negative sample sampling.


$$\mathrm{\backslash [}L=\underset{\left(h,r,t\right)\in T}{\sum }\;\underset{\left(h^{\prime },r^{\prime },t^{\prime }\right)\in T^{\prime }}{\sum }\;\left(L_{1}\left(h,r,t\right)+\alpha_{1}\underset{p\in P\left(h,t\right)}{\sum }\;L_{2}\left(p,r\right)+\alpha_{2}\underset{r_{e}\in R\left(r\right)}{\sum }\;L_{3}\left(r,r_{e}\right)\right),\mathrm{\backslash ]}$$ (12)

*T* represents the positive triples, while *T*' denotes the corresponding negative samples. *R*(*r*) includes relations derived from *r* through rule *R*_1_. *P* (*h, t*) represents the set of paths between (*h, t*), with P being an individual path. *L*_1_*, L*_2_, and *L*_3_ correspond to the marginal loss functions for entity triples (*h, r, t*), path-relation pairs (*p, r*), and relation pairs (*r, r*_e_), respectively. Specifically, the marginal loss function for entity triples is given as follows:


$$L_{1}\left(h,r,t\right)=\max\left(0,-\log\;\;\sigma \;\;\left({\;\;\gamma \;\;}_{1}-E\left(h,r,t\right)\right)-\underset{i=1}{\overset{n}{\sum }}\;\frac{1}{k}\log\;\;\sigma \;\;\left(E\left(h^{\prime },r,t^{\prime }\right)-{\;\;\gamma \;\;}_{1}\right)\right)$$ (13)

where γ_1_ represents a fixed boundary value, and σ denotes the sigmoid function. During the triplet negative sampling process, a self-adversarial negative sampling approach is employed to address the inefficiency associated with standard negative sampling. Specifically, negative triples are sampled as follows:


$$p\left(h_{j}^{\prime },r,t_{j}^{\prime }|\left\{\left(h_{i},r_{i},t_{i}\right)\right\}\right)=\frac{\exp\alpha \;f_{r}\left(h_{j}^{\prime },t_{j}^{\prime }\right)}{\sum \{i\exp\alpha \;f_{r}\left(h_{i}^{\prime },t_{i}^{\prime }\right)},$$ (14)

α represents the sampling temperature. To reduce costs, probabilities are employed as weights for negative samples. The final loss function is defined as follows:


$$L_{1}\left(h,r,t\right)=\max\left(0,-\log\sigma \left(\gamma_{1}-E_{1}\left(h,r,t\right)\right)-\sum_{i=1}^{n}p\left(h^{\prime },r,t^{\prime }\right)\log\sigma \left(E_{1}\left(h^{\prime },r,t^{\prime }\right)-\gamma_{1}\right)\right),$$ (15)

$$L_{2}\left(p,r\right)=\max\left(0,{\;\;\gamma \;\;}_{2}+E_{2}\left(p,r\right)-E_{2}\left(p^{\prime },r^{\prime }\right)\right),$$ (16)

$$L_{3}\left(r,r_{e}\right)=\max\left(0,{\;\;\gamma \;\;}_{3}+\;\;\beta \;\;E_{3}\left(r,r_{e}\right)-E\left(r,r^{\prime }\right)\right).$$ (17)

where *γ*_*2*_ and *γ*_*3*_ > 0 are hyperparameters, and *β* represents the confidence level associated with *r* and *r*_*e*_. The Adam optimizer [26] was employed, and hyperparameters were fine-tuned on the validation set. To maintain training efficiency, the path length was restricted to a maximum of 3 steps.

## 4. Experiments

### 4.1. Experimental setting

#### Datasets and rules.

The proposed model was evaluated on three benchmark datasets: FB15K and FB15K-237 from Freebase, and WN18 from WordNet. FB15K-237, a subset of FB15K, excludes inverse relations and emphasizes modeling symmetric/antisymmetric properties and compositional patterns for link prediction. Table 2 provides detailed dataset statistics, including the number of entities, relationships, and triples in the training, validation, and testing sets. The performance of the proposed method was compared with baseline approaches on entity prediction tasks for KG completion.

**Table 2:** Data statistics of the three datasets used in the experiment.

Dataset	Relationships	Entities	Training	Validation	Testing
FB15K	1,345	14,951	483,142	50,000	59,071
FB15K-237	237	14,541	272,115	17,535	20,466
WN18	18	40,943	141,442	5,000	5,000

#### Evaluation protocols.

Test triples, along with candidate triples not included in the training, validation, or test sets, were organized for evaluation. The candidate triples were generated by modifying the subject or object, yielding (*h′, t, r*) or (*h, t′, r*). The primary metrics used for evaluation are Mean Rank (MR), Mean Reciprocal Rank (MRR), and Hits@n, which represents the proportion of correct entities ranked in the top *n* predictions. The goal is to achieve a lower MR, higher MRR, and higher Hits@10. To accomplish this, reconstructed triples are scored using the following function:


$$Q\left(h,r,t\right)=∥hr-t∥+{\;\;\alpha \;\;}_{1}\underset{p\in P\left(h,t\right)}{\sum }\;R\left(p|h,t\mathrm{\backslash rightleft}(\underset{{\;\;\mu \;\;}_{1}\in B\left(p\right)}{\prod }\;{\;\;\mu \;\;}_{1}\right)∥C\left(p\right)-r∥$$ (18)

As shown in Eq. 18, rule R_2_ is utilized to amalgamate paths during the testing phase.

#### Baselines.

For the purpose of evaluating the efficacy of our approach, a selection of models for knowledge graph completion serves as benchmarks. These benchmarks are divided into three distinct categories: (1) embedding models that concentrate exclusively on triples, such as TransE, DistMult, ComplEx, and RotatE; (2) a path-based model, PTransE; and (3) a rule-enhanced model, RPJE. We have adopted the optimal results reported in their respective original studies and implemented RotatE and RPJE using their authentic source codes.

#### Experimental settings.

To ensure a fair and equitable comparison, the following configurations were employed: (1) The real and imaginary parts of the entity embeddings were initialized randomly, whereas relation embeddings were initialized uniformly across the interval from 0 to 2π. Regularization was not included, as a fixed margin γ_1_ effectively mitigated overfitting. In line with the standard baseline setups, the learning rate was set to 0.0001, and γ_2_ was fixed at 1.

A furthermore, a grid search was performed to determine the most effective hyperparameters. These included embedding dimensions *d* spanning from 125 to 1000, Furthermore, a grid search was performed to determine the most effective hyperparameters. These included embedding dimensions *d* spanning from 125 to 1000, self-adversarial sampling temperatures *α* of 0.5 and 1.0, a fixed margin γ_1_ set at 3, and γ_3_ values ranging from 0.5 to 3.5. We utilized the best results reported in the original studies and implemented RotatE and RPJE using their respective source codes.

### 4.2. Quality assessment of rules

In the realm of rule mining, Standard Confidence (SC) is a widely adopted metric for assessing the quality of extracted rules., measuring how often the rule head occurs if the rule body holds. In order to fairly compare the rule quality of different methods, we compute the average SC scores of each method on the top K (K = 50, 100, 200, 500) rules and conduct experiments on the FB15K-237 dataset (as shown in Table 3). The rules for NeuralLP [32] and DRUM [33] were derived from their original implementations. Taking the FB15K-237 dataset as an example, the table presents the results of various rule extraction models. The findings reveal that Ruleformer achieves a significantly higher standard confidence for subgraph-based parsing rules compared to other methods when dealing with triplets that share the same relationship.

**Table 3:** Avg. confidence on FB15K-237, by rule length T.

Methods	FB15K-237		
	TOP		
	50	200	500
Neural-LP(T^*^ = 2)	.020	.044	.033
Neural-LP(T = 3)	.020	.031	.034
DRUM(T = 2)	.058	.036	.048
DRUM(T = 3)	.020	.039	.027
Ruleformer(T = 2)	.241	**.338**	**.310**
Ruleformer(T = 3)	**.313**	.322	.282

* T represents the depth of the inference step.

Higher confidence levels indicate that more reliable and valid rules are being utilized in the process. Additionally, it has been observed that under the same model, rules with a length of 2 are more effective, likely because longer paths often lead to less accurate compositions. As a result, a path step size of 2 was chosen as the optimal configuration for the subsequent results.

### 4.3. Link prediction

Link prediction is a cornerstone task in knowledge graph (KG) embedding, focused on forecasting missing or potential connections between entities within a KG. It is pivotal for KG completion, knowledge base reasoning, and a variety of applications that necessitate inference or the uncovering of missing facts. A comparative analysis was performed between RotatE and several leading models, with RotatE and RPJE being chosen as benchmarks. Their published results were directly utilized due to the common evaluation dataset employed. Table 4 displays our findings on the FB15k, WN18, and FB15K-237 datasets, clearly showing that TP-RotatE surpasses all other cutting-edge models in performance.

**Table 4:** Link prediction results on FB15k, WN18, and FB15K-237.

Models	FB15K			WN18			FB15K-237		
	MR	MRR	H@10	MR	MRR	H@10	MR	MRR	H@10
**TransE**	-^*^	.463	.749	–	.495	.943	357	.294	.465
**DistMult**	42	.798	.893	655	.797	.946	254	.241	.419
**ComplEx**	–	.692	.840	–	.941	.947	339	.247	.501
**PTransE**	54	.679	.834	–	.890	.945	302	.363	.526
**RPJE**	**40**	.816	.903	–	.946	.951	207	.470	.625
**RotatE**	**40**	.797	.884	**309**	.949	.959	177	.338	.533
**TP-RotatE**	42	**.827**	**.912**	318	**.951**	**.960**	**169**	**.484**	**.642**

* Missing values indicate unreported scores.

Bold highlights the best score, underline marks the second best.

The proposed TP-RotatE method was initially subjected to a rigorous evaluation against various baselines for the task of link prediction on the FB15K dataset. The following insights can be gleaned from the results presented in Table 4: (1) TP-RotatE outperforms the baseline models, with the majority of these improvements being statistically significant, demonstrating its robustness and reliability. (2) Notably, TP-RotatE outshines RPJE across all evaluated metrics, signifying its superiority in leveraging head entity rules extracted via a neural network approach. This leads to higher accuracy in path composition and enhanced path embeddings. (3) TP-RotatE shows a 6.3% and 4.4% improvement in Mean Reciprocal Rank (MRR) compared to the rule-based baseline RotatE. This underscores the effectiveness of incorporating rules to preserve more semantic information and to strengthen the integration of paths. Furthermore, experiments conducted on the FB15K-237 dataset reveal that while TP-RotatE performs comparably to RPJE, it exhibits slight advantages on most metrics. This suggests that TP-RotatE is more refined and efficient in integrating paths and rules. On the FB15K-237 dataset, where inverse relationships are not present, efficient rule mining serves as a valuable adjunct to link prediction, bolstering semantic relevance.

### 4.4. Experimental results by relation category

In knowledge graph (KG) link prediction, the diversity of relationship types encapsulates specific interactions and semantic linkages between entities, which significantly impact the precision of predictions. Therefore, the analysis of link prediction outcomes is segmented according to these varying relationship types. As defined by Bordes et al. [10], relationships within a knowledge base can be categorized based on their mapping characteristics (1-1, 1-N, N-1, N-N). Table 5 details the comparative performance of our model and several selected baselines across different types of relationships, with the evaluation conducted using the FB15K dataset.

**Table 5:** Results on FB15K.

Models	Head Prediction (Hits@10)				Tail Prediction (Hits@10)			
	1-1	1-N	N-1	N-N	1-1	1-N	N-1	N-N
TransE	.437	.657	.182	.472	.437	.197	.667	.500
TransH	.668	.876	.287	.645	.655	.398	.833	.672
ComplEx	.939	.969	.692	.893	.938	.823	.952	.910
TransG	.930	.960	.625	.868	.928	.681	.945	.888
PTransE	.910	.928	.609	.838	.912	.740	.889	.864
RPJE	.942	.965	.704	.916	.941	.839	.953	.933
RotatE	.922	.967	.602	.893	.923	.713	.961	.922
TP-RotatE	**.949**	**.971**	**.743**	**.931**	**.950**	**.852**	**.970**	**.961**

1) The proposed model demonstrates a consistent and significant outperformance across all baseline methods within various relational categories. It achieves an average improvement of 2.1% in head entity prediction compared to RPJE, with particularly remarkable enhancements of 3.6% observed in many-to-one and many-to-many relationships. In the case of tail entity predictions, the model shows an average improvement of 1.8%, with more substantial gains of 2.4% in many-to-one and many-to-many scenarios.
2) These results suggest that the model is equally effective on non-injective relationships as it is on one-to-one relationships. This highlights the significance of integrating additional entity rules to retain semantic information, which markedly improves the accuracy of entity prediction.

### 4.5. Ablation study

To assess the importance of TP-RotatE components, ablation experiments were conducted on FB15K by removing paths and extracted rules. As shown in Table 6, tp-RotatE-p and tp-RotatE-rul represent the TP-RotatE model without paths and without logical rules, respectively. Evidently, the removal of any component results in a decline in model performance.

**Table 6:** Ablation studies by removal paths and logical rules.

Models	MR		MRR	Hits@10%	
	Raw	Filtered	Filtered	Raw	Filtered
TP-RotatE- p	186	40	0.811	50.2	88.4
TP-RotatE- rul	217	49	0.714	44.3	81.8
TP-RotatE	**183**	**38**	**0.824**	**51.4**	**90.9**

### 4.6. Case study

**Fig 3:**
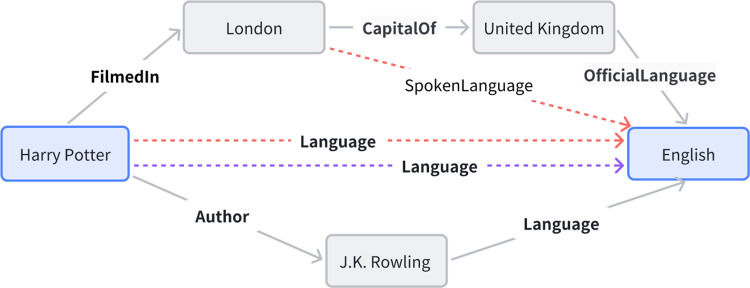
An example of the explainable entity prediction.

Consider an entity prediction task as shown in Fig 3, given that the head entity is *Eiffel Tower* and the relation is *localisedIn*. our goal is to predict the tail entity. Our model TP-RotatE yields the result: *France*. Here is a detailed description of the reasoning process.

For simple two-step paths, the embedding model in the model can directly activate ‘*locatedIn*(*x*, *y*) = (*situatedIn*(*x*, *z*) ∧ *capitalof*(*z*, *y*))’ by successive rotation actions, which in turn predicts the tail entity *France*.

For the three-step path of the complex relationship, the model first determines the association of *Eiffel Tower* with *FrenchCulture* by the rule ‘*CulturalSignificance* (*architecturalStyle, associatedWith*)’, and then determines the association of *Eiffel Tower* with *FrenchCulture* by the rule ‘*locatedIn* (*FrenchCulture, France*)’ determines that *FrenchCulture* is located in *France*. This intermediate combination result *CulturalSignificance* is further used to achieve the predicted result *France* for the tail entity through embedding on the reconstructed path of the inclusion relation *FrenchCulture*. The three-step path effectively simplifies the combination of complex relations through the model’s path fusion technique, and rule mining also provides more possibilities for combining paths.

### 4.7. Model performance analysis on different types of relationships

**Fig 4:**
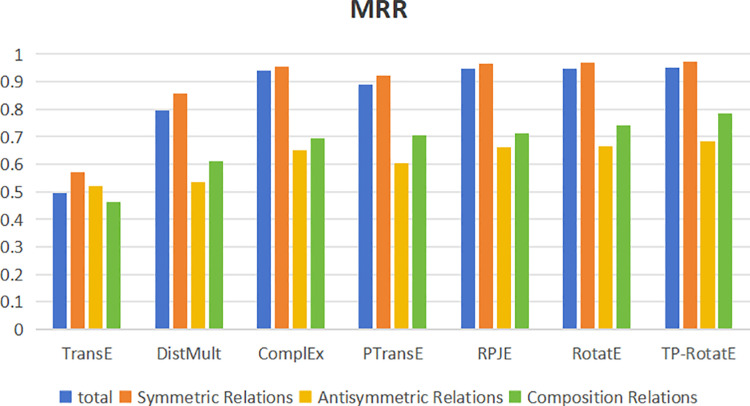
MRR comparison across different relationship types.

**Fig 5:**
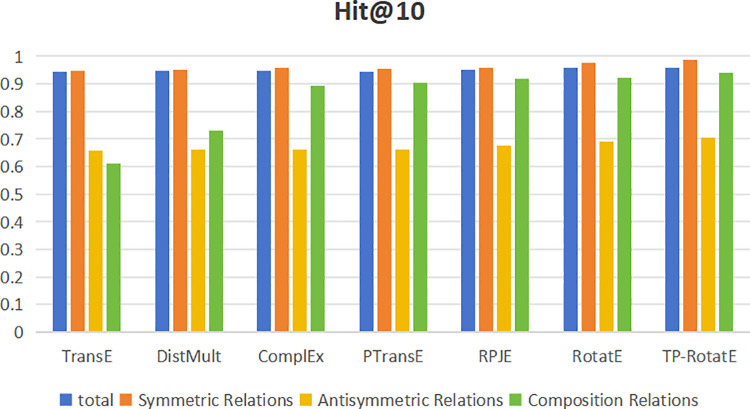
Hit@10 comparison across different relationship types.

To conduct an in-depth analysis of how various models handle different relationship types—including symmetric, antisymmetric, and compositional relations—we evaluate their performance using Mean Reciprocal Rank (MRR) and Hits@10. The results, visualized in bar charts (Figs 4 and 5), offer a comprehensive comparison across models. Below, we summarize the key findings: Across all evaluation metrics, TP-RotatE consistently outperforms other models, achieving an MRR of 0.951 and H@10 of 0.96. These results highlight its superior capability in the knowledge graph completion task. As shown in Fig 4, TP-RotatE maintains a leading position in modeling symmetric relations (MRR = 0.971), surpassing RotatE (0.968) and RPJE (0.966). This suggests that TP-RotatE effectively captures relational equivalence, making it particularly well-suited for learning symmetric dependencies. For antisymmetric relations, TP-RotatE demonstrates a clear advantage with an MRR of 0.683, outperforming RotatE (0.664) and ComplEx (0.649). These results indicate that TP-RotatE is more adept at distinguishing directional dependencies, thereby enhancing predictive accuracy when modeling antisymmetric relations. When handling compositional relationships, TP-RotatE once again achieves the highest performance (MRR = 0.786), outperforming RPJE (0.713) and RotatE (0.741). This suggests that TP-RotatE exhibits superior generalization capabilities in learning complex relational structures.

As depicted in Fig 5, the H@10 trends align closely with the MRR results, further reinforcing TP-RotatE’s superior adaptability across different relationship types. These findings suggest that TP-RotatE is not only effective in general knowledge graph completion but also excels at modeling diverse relational structures. The empirical analysis demonstrates that TP-RotatE consistently achieves state-of-the-art performance across symmetric, antisymmetric, and compositional relations. These results underscore its enhanced ability to capture intricate relational patterns, further validating its effectiveness in knowledge graph completion tasks.

## 5. Conclusion and future work

This study introduces TP-RotatE, a novel knowledge graph (KG) representation learning model that represents entities as complex vectors and relations as rotations within the complex vector space. The model seamlessly integrates path information and contextual rules to address complex relation types, enabling a more holistic representation of KG relationships. Within the model, rules of lengths 1 and 2 are extracted using Ruleformer by consolidating context information around the head entity. These rules are then incorporated into the embedding framework, alongside relationship path information, to resolve additional relationship combinations. The integration of these rules enhances the generation of relational paths, thereby improving the precision of reasoning. The model adeptly captures hidden relationships by considering a multitude of relationship types. Experimental results indicate that TP-RotatE outperforms all baseline models, validating the effectiveness of employing deep learning for rule extraction and the integration of relationship types and paths to enhance KG prediction accuracy.

### 5.1. Limitations

Despite its promising performance, TP-RotatE has several limitations: (1)Local Context Dependence: The reasoning process primarily focuses on local contextual information. However, more complex reasoning tasks may require a broader global perspective to provide richer semantic understanding. Enhancing the model’s global perception could improve its ability to handle more intricate inference tasks. (2) Long-Path Information Representation: The current model may struggle to effectively learn from longer relational paths, leading to under-representation of global knowledge dependencies. This limitation impacts the model’s ability to capture deeper relational structures in knowledge graphs.

### 5.2. Future work

To address these limitations, future research will focus on the following directions: (1) Enhancing Global Awareness: We aim to explore strategies for integrating global knowledge representations to improve semantic understanding in complex reasoning tasks. This includes developing hybrid architectures that combine local path-based inference with global KG structural information. (2) Scalability Optimization: Future work will extend TP-RotatE to large-scale KGs, optimizing computational efficiency to reduce resource consumption. Techniques such as model pruning, efficient sampling strategies, and distributed learning will be explored to enhance scalability. (3) Improving Long-Path Representation: More advanced sequence modeling techniques will be introduced to handle longer and more complex paths, ensuring better knowledge propagation across distant entities. Potential solutions include leveraging transformer-based architectures or recurrent relational reasoning mechanisms.

By addressing these limitations, we aim to further enhance the applicability and robustness of TP-RotatE in real-world KG reasoning tasks.
